# Cold atmospheric plasma induces apoptosis in human colon and lung cancer cells through modulating mitochondrial pathway

**DOI:** 10.3389/fcell.2022.915785

**Published:** 2022-07-26

**Authors:** Yanhong Wang, Xinyu Mang, Xuran Li, Zhengyu Cai, Fei Tan

**Affiliations:** ^1^ Shanghai Fourth People’s Hospital, School of Medicine, Tongji University, Shanghai, China; ^2^ Department of Biochemistry and Molecular Biology, State Key Laboratory of Medical Molecular Biology, Institute of Basic Medical Sciences Chinese Academy of Medical Sciences, School of Basic Medicine Peking Union Medical College, Beijing, China; ^3^ Tongji University Cancer Center, Shanghai Tenth People’s Hospital, School of Medicine, Tongji University, Shanghai, China; ^4^ The Royal College of Surgeons in Ireland, Dublin, Ireland; ^5^ The Royal College of Surgeons of England, London, United Kingdom

**Keywords:** cold atmospheric plasma, apoptosis, reactive oxygen species, mitochondria, caspase

## Abstract

Cold atmospheric plasma (CAP) is an emerging and promising oncotherapy with considerable potential and advantages that traditional treatment modalities lack. The objective of this study was to investigate the effect and mechanism of plasma-inhibited proliferation and plasma-induced apoptosis on human lung cancer and colon cancer cells *in vitro* and *in vivo*. Piezobrush^®^ PZ2, a handheld CAP unit based on the piezoelectric direct discharge technology, was used to generate and deliver non-thermal plasma. Firstly, CAP_PZ2_ treatment inhibited the proliferation of HT29 colorectal cancer cells and A549 lung cancer cells using CCK8 assay, caused morphological changes at the cellular and subcellular levels using transmission electron microscopy, and suppressed both types of tumor cell migration and invasion using the Transwell migration and Matrigel invasion assay. Secondly, we confirmed plasma-induced apoptosis in the HT29 and A549 cells using the AO/EB staining coupled with flow cytometry, and verified the production of apoptosis-related proteins, such as cytochrome c, PARP, cleaved caspase-3 and caspase-9, Bcl-2 and Bax, using western blotting. Finally, the aforementioned *in vitro* results were tested *in vivo* using cell-derived xenograft mouse models, and the anticancer effect was confirmed and attributed to CAP-mediated apoptosis. The immunohistochemical analysis revealed that the expression of cleaved caspase-9, caspase-3, PARP and Bax were upregulated whereas that of Bcl-2 downregulated after CAP treatment. These findings collectively suggest that the activation of the mitochondrial pathway is involved during CAP_PZ2_-induced apoptosis of human colon and lung cancer cells *in vitro* and *in vivo*.

## Introduction

Cancer is a leading cause of death worldwide, accounting for nearly 10 million deaths in 2020 (Ferlay et al., 2021). Colon cancer and lung cancer are among the top three cancers in terms of number of new cases (1.9 and 2.2 million) and number of cancer death (0.9 and 1.8 million), respectively. Depending on the cancer staging, the main treatment options for these prevalent and lethal malignancies include, but are not limited to, surgery, chemotherapy, and radiation therapy. However, the prognosis of colon and lung cancer has never been satisfying, especially for patients with metastatic lesions. As new optional approach, targeted therapy and immunotherapy have successfully prolonged overall survival for these patients, collectively changing the treatment paradigm of colon cancer and lung cancer. Nonetheless, these newer treatment modalities have limitations. Therefore, there is a constant drive to seek more versatile and adaptable oncotherapy.

As a new biomedical technology, cold atmospheric plasma (CAP) is an ionized non-thermal gas mixture composed of various reactive oxygen species (ROS), reactive nitrogen species and ultraviolet photons (Fridman et al., 2008; Ratovitski et al., 2014; von Woedtke et al., 2019). CAP has demonstrated great potential in cancer treatment (Arndt et al., 2013; Adhikari et al., 2019). Since the killing effect of CAP on melanoma was first reported, the research on the application of CAP in oncology has progressed rapidly (Binenbaum et al., 2017). Many studies have shown that CAP can inhibit tumor growth and promote tumor death in various malignant tumor cells, including but are not limited to, hepatocellular carcinoma cells, melanoma cells, prostate cancer cells, cervical cancer cells, cervical squamous cell carcinoma cells, oral squamous cell carcinoma cells, osteosarcoma cells, multiple myeloma cells, lymphoma cells (Walk et al., 2013; Gumbel et al., 2016; Cheng et al., 2017; Yan et al., 2017). CAP can not only selectively induce tumor cell apoptosis, but also inhibit tumor cell migration and invasion (Xu et al., 2017). In addition, CAP enhances the sensitivity of tumor cells to chemotherapeutic drugs (Ishaq et al., 2015), achieving antitumor effect with high efficiency and low toxicity. Meanwhile, some studies have confirmed that the synergistic effect of CAP and nanoparticles renders stronger antineoplastic ability (Aryal and Bisht, 2017). However, the exact mechanism of CAP’s oncological advantage is not completely elucidated, and it might be related to its active components, such as ultraviolet, charged particles, and chemically active elements (Volotskova et al., 2012).

CAP is a type of low-temperature plasma, which produces various free radicals that are suitable for medical treatment (Wang et al., 2013). CAP damages cancer cells intracellularly, such as destroying DNA and mitochondria leading to apoptosis of the treated cells (Kim et al., 2010; Ahn et al., 2011). These changes are mainly due to the accumulation of intracellular ROS in cancer cells, resulting in mitochondrial dysfunction, sub-G1 arrest and DNA damage (Lee et al., 2016). Compared to normal cells, cancer cells are more likely to accumulate ROS because of the increased production rate of ROS, which promote several aspects of cancer cell proliferation and metastasis (Liou and Storz, 2010). According to previous studies, the production of ROS may be the main underlying mechanism of CAP-induced apoptosis (Wang et al., 2019).

We were the first to use the newly developed Piezobrush compact handheld plasma device, which is based on the piezoelectric direct discharge (PDD) technology, on cancer treatment. The primary objective of this study was to evaluate the influence of the above plasma platform, CAP_PZ2_, on human colon and lung cancer cells, highlighting the effect of CAP_PZ2_ treatment on the proliferation, migration/invasion, and apoptosis of HT29 and A549 cells *in vitro* and *in vivo*. The secondary objective was to explore the potential molecular mechanism of plasma-induced apoptosis in the colorectal and pulmonary cancer model. This provides new insights for the clinical treatment of colon cancer and lung cancer, potentially serving as a novel oncotherapy.

## Materials and methods

### Cell culture

**FIGURE 1:**
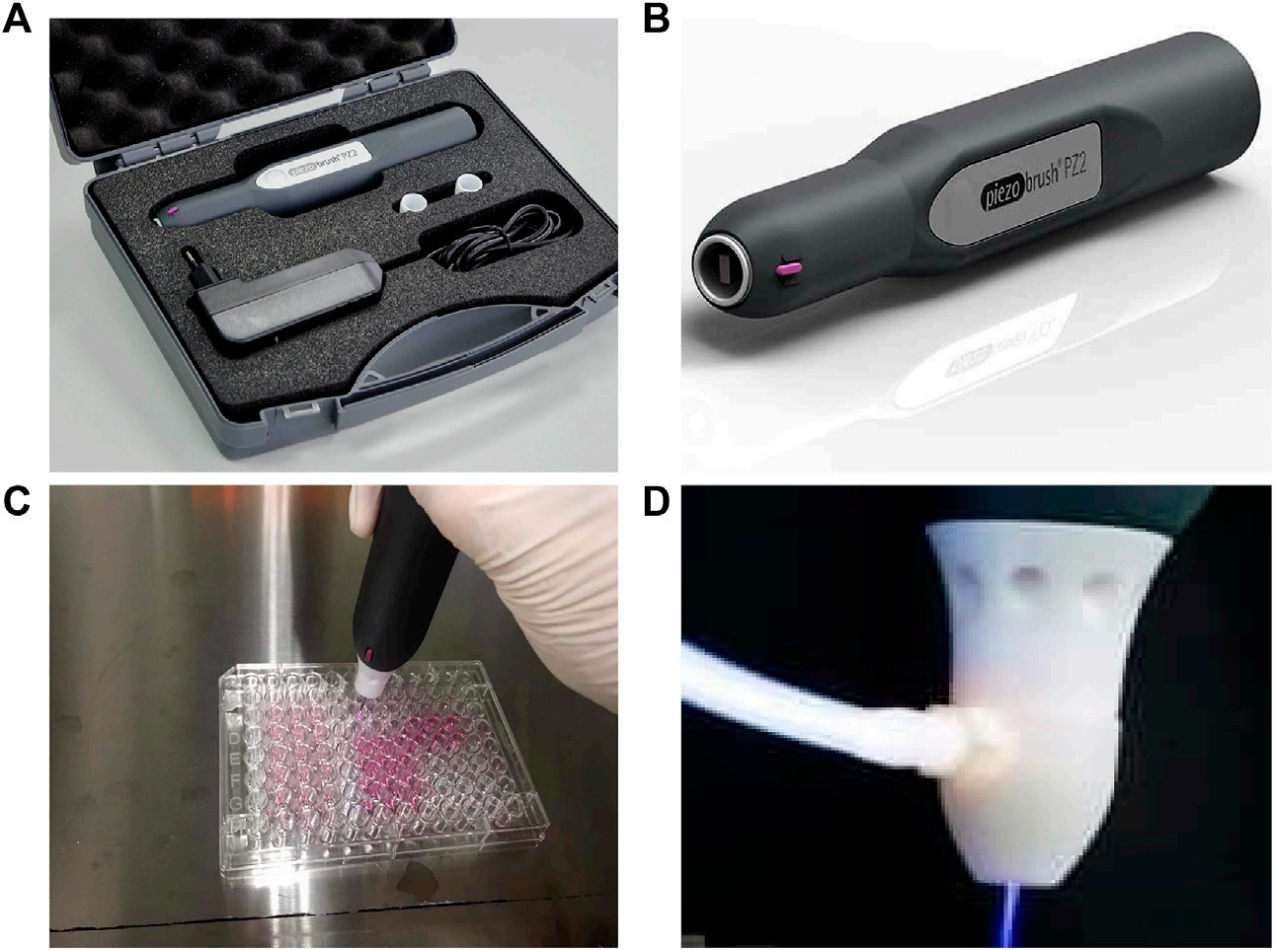
Illustration of the CAP device, Piezobrush^®^ PZ2, used in this study. **(A,B)** Content and portability of the CAP_PZ2_ kit. **(C)** Cellular treatment using the handheld plasma device. **(D)** Close-up of the plasma nozzle in the operating mode.

Human colorectal adenocarcinoma HT29 cells and human lung adenocarcinoma A549 cells were kindly provided by Professor Zhenyu Cai (Tongji University, Shanghai, China). These cells were cultured at 37°C with 5% CO_2_ in The Piezobrush® PZ2 model was used (Tinsley and Harris, 2020) and has been conceived to be an efficient handheld unit (Figures 1A,B). The CAP_PZ2_ device, at a maximum power consumption of 30 W, generated cold active plasma with an a nozzel temperature of <50°C. The plasma nozzle was placed 10 mm above the treated surface, with treatment duration of 15, 30, 60 and 120 s (Figures 1C,D). In order to examine the effect of CAP_PZ2_ treatment on various tumor cells, we performed a direct plasma treatment on the HT29 and A549 cells without coverage by the cell culture medium. This contrasts with indirect plasma treatment used in some other studies, e.g., plasma-activated medium (PAM).

A complete cell growth medium containing DMEM (Corning, United States), 10% fetal bovine serum (FBS) and 1% penicillin and streptomycin (Gibco, United States). The cells were cultured to a confluence of 85%–90% and then subcultured using 0.25% trypsin (Gibco, United States). The cells were gathered in the exponential growth phase.

### Cold atmospheric plasma treatment

The plasma device used in this study is an ambient air-dependent device that was designed and developed by Relyon Plasma GmbH (Regensburg, Germany).

### Cell counting kit-8 assay

The inhibitory effect of CAP_PZ2_ on HT29 and A549 cell proliferation was measured using Cell Counting Kit-8 (CCK8, Dojindo Laboratories, Japan) assay. CAP_PZ2_ was attached to a small stereotaxic apparatus with its tip adjusted to a height of 10 mm above the fluid level of cell culture. CAP_PZ2_ was then administered for designated time period. After 24 h of incubation, the cells were treated by CAP_PZ2_ (0, 15, 30, 60 and 120 s) in 96 well-plates (3× 10^3^ cells/well) and incubated for a further 24 h. Following CAP_PZ2_ treatment, CCK8 assay was performed according to manufacturer’s instructions at 37°C for 2 h. The absorbance was read at 450 nm using an ELISA microplate reader (Molecular Devices, Sunnyvale, United States). All experiments were performed in triplicates.

### Hoechst fluorescent staining

The HT29 and A549 cells were seeded in 12-well plates and exposed to CAP_PZ2_ treatment for 15, 30 and 60 sec. Following incubation of 24 h, the cells were stained with Hoechst 33342 (Beyotime Biotechnology, China) at 37°C for 30 min, and then washed twice with phosphate-buffered saline (PBS) for three minutes each. Nuclear morphological changes were observed using an Olympus fluorescent microscopy.

### Transmission electron microscopy

The HT29 and A549 cells were treated with CAP_PZ2_ (30 s) at 37°C for 24 h and then collected for electron microscopy. Firstly, the cell precipitates were fixed in 5% glutaraldehyde for 30 min, and then placed in 1% osmium tetroxide and 0.1 mol/L sodium cacodylate (pH 7.4) for 1 h. The cell precipitates were desiccated in a graded series of acetone and embedded with EPON-812. Ultra-thin sections were prepared, double-stained with uranium and plumbum, and observed under transmission electron microscope using x10,000 magnification. Meanwhile, untreated cells were used as control.

### Transwell migration and invasion assay

Cell motility and invasiveness were measured using the Transwell assay (Millipore, Billerica, United States). The HT29 and A549 cells were seeded into 6-well plates at a density of 2 × 10^5^ cells/well and treated with the CAP_PZ2_ (0, 30 and 60 s, respectively). For the migration assay, approximately 200 μL of 1×10^5^ HT29 or A549 cells were seeded into the upper chamber containing serum-free medium, while the bottom of the chamber contained 800 μL of DMEM with 10% FBS. For the invasion assay, the chamber was coated with 100 μL of Matrigel (BD Biosciences, United States), and the subsequent steps were similar to those of the migration assay. After 24–48 h of incubation, the medium was discarded. Cells that had invaded the lower surface of the membrane were fixed in Methanol for 30 min and stained with 0.1% crystal violet for 30 min. Migrated and invaded cells in random fields were examined and counted using Olympus microscopy at ×200 magnification.

### RNA extraction and qRT-PCR

Four metastasis-associated messenger RNA (mRNA) gene expression (*MMP9*, *MMP2*, *VEGF*, *MTDH*) was detected regarding different times of CAP_PZ2_ treatment. Briefly, HT29 and A549 cells were seeded in 6-well plate and incubated 24 h. CAP_PZ2_ treatment homogeneity was then conducted with 30 and 60 s and cells were allowed growth for another 24 h. Total RNA was extracted from cell using Trizol reagent (Invitrogen) according to the manufacturer’s instruction. The NanoDrop1000 Spectrophotometer (ThermoScientific) was employed to quantify the amount of RNA samples. Specifically, 1 μg of total RNA was utilized for reverse transcription using RevertAid First Strand cDNA Synthesis kit (Thermo Fisher Scientific). The qRT-PCR was performed using the ABI 7500 Real-Time PCR System (Applied Biosystems) to verify gene expression in various cells. qRT-PCR was performed using PowerUp SYBR Green Master Mix (Applied Biosystems). The primer sequences are forMMP9: forward, 5′-AGA​CCT​GGG​CAG​ATT​CCA​AAC-3′ and reverse, 5′-CGG​CAA​GTC​TTC​CGA​GTA​GT-3′; for MMP2: forward, 5′-GAT​ACC​CCT​TTG​ACG​GTA​AGG​A-3′ and reverse, 5′-CCT​TCT​CCC​AAG​GTC​CAT​AGC-3′; for VEGF: forward, 5′-AGG​GCA​GAA​TCA​TCA​CGA​AGT-3′ and reverse, 5′-AGG​GTC​TCG​ATT​GGA​TGG​CA-3′; for MTDH: forward, 5′-AAG​CAG​TGC​AAA​ACA​GTT​CAC​G-3′ and reverse, 5′-GCA​CCT​TAT​CAC​GTT​TAC​GCT-3′. The cells without CAP_PZ2_ treatment were selected as control.

### Detection of apoptosis

The HT29 and A549 cells were seeded into 6-well plates at a density of 2 × 10^5^ cells/well and exposed to CAP_PZ2_ treatment for 30 and 60 s. After treatment for 24 h, the cells were detached and stained using PE Annexin V apoptosis detection kit I (BD Biosciences, San Jose, CA, United States), according to manufacturer’s instructions. In brief, the HT29 or A549 cells were collected after CAP_PZ2_ treatment, washed with cold PBS and incubated at room temperature with PE Annexin V and 7-AAD for 15 min in darkness. Flow cytometry analysis was performed within 1 h. The HT29 or A549 cells were separated using the BD LSRFortessa™ flow cytometer (BD Biosciences). At least 20,000 cells were analyzed from each treatment group.

### Acridine orange and ethidium bromide cell staining

The acridine orange and ethidium bromide (AO/EB) (BestBio, China) staining was used to distinguish viable cells from apoptotic and necrotic cells. The HT29 and A549 cells (1 × 10^5^ cells/well) were inoculated in 24-well plates and allowed to adhere for 12 h. These cells were then treated using CAP_PZ2_ (30 and 60 s) for 24 h. The HT29 and A549 cells were harvested, centrifuged, resuspended in 100 μl of culture medium, and stained with 5 μl AO and EB, respectively. The cell suspension was immediately examined using fluorescence microscopy (Olympus), and the obtained images were analyzed accordingly. Cells with uniformly bright green nuclei and an organized structure were considered as viable cells, those with orange to red nuclei, condensed to fragmented chromatin and green cytoplasm as apoptotic cells, and those with uniformly orange to red nuclei, an organized structure and red cytoplasm as necrotic cells.

### Measurement of reactive oxygen species

The HT29 and A549 cells were seeded in 6-well culture plates at a density of 2 × 10^5^ cells/well and treated using CAP_PZ2_ at 0, 30 and 60 s for 24 h. The cells were harvested, washed, and resuspended in serum-free DMEM. The resuspended cells were stained with 10 μM fluorescent dye DCFH-DA (Beyotime Biotechnology, China) at 37°C for 20 min, and rewashed 3 times by PBS. The fluorescence from the HT29 and A549 cells were measured using the BD LSRFortessa™ flow cytometer (BD Biosciences). At least 10,000 cells were analyzed from each treatment group.

### Western blot

The HT29 and A549 cells were cultured in 6-well plates at a density of 2 × 10^5^ cells/well and treated using CAP_PZ2_ (0, 30 and 60 s, respectively). After 24 h, cells were harvested and washed with PBS solution. The whole-cell lysates were prepared using RIPA lysis buffer (Solaibao, Beijing, China) containing cOmplete™ Protease Inhibitor Cocktail tablets (Roche, Indianapolis, IN). Protein concentrations were quantified using a BCA protein assay kit (Solaibao, Beijing, China) according to the instructions. For the western blotting analysis, 40 μg of protein from each sample was loaded into 10%–15% SDS-PAGE and transferred onto a PVDF membrane (Immobilon-P, CA, United States). The membranes were incubated overnight at 4°C with suitably diluted primary antibodies against cytochrome c, cleaved poly (ADP-ribose) polymerase (cleaved-PARP), cleaved caspase-3, cleaved caspase-9, Bcl-2 or Bax (Cell Signaling Technology, United States). The signals were developed using an enhanced chemiluminescence (ECL) kit (Immobilon-P, CA, United States), and the bands visualized using the ChemiDoc™ Touch Image system (Bio-Rad).

### Nude mice xenograft model

Thirty-two female BALB/c nude mice were purchased from Zhejiang Vital River Laboratory Animal Technology Co., Ltd. (Zhejiang, China, SCXK (ZHE) 2019-0001, Animal Certificate No.: 20211111Abzz0619000810), and housed under controlled temperature (22–24°C) and pathogen-free conditions at the Experimental Animal Center, School of Medicine, Tongji University. All procedures were performed aseptically in accordance with the standard guidelines under a protocol approved by the Experimental Animal Ethics Committee of Tongji University. The HT29 or A549 cells (5×10^6^ cells) were inoculated subcutaneously into the right anterior armpit of nude mice when the mice turned 6 weeks old. When the tumor size reached approximately 50 mm^3^, these mice were randomly divided into control and CAP_PZ2_ groups (*n* = 8 each). The animals in the CAP_PZ2_ group were treated for 2 min each time and thrice weekly until the end of the study. The tumor volume (V) was determined by measuring the length (a) and width (b) every 3 or 4 days and calculated using the following formula: V (mm^3^) = ½ x ab^2^. All mice were sacrificed, and tumors were collected and weighed 24 h after the last treatment.

### Histology and immunostaining

Mouse tumours were resected, fixed in 10% neutral buffered formalin overnight at 4°C, and paraffin embedded. Five-micrometre sections were processed for hematoxylin and eosin (HE) or mmunohistochemical (IHC) staining per standard protocol. For immunohistochemistry staining, tissue sections were first deparaffinized in xylene and rehydrated in graded ethanols. Then, the tumor slides were immersed in methanol containing 0.1% hydrogen peroxide for 10 min to block endogenous peroxidase activity. Antigen repair was performed by treating the slides with pH 6.0 citrate buffer for 20 min in a microwave oven. The cytochrome *c* (Cell Signaling Technology, 1:100), cleaved PARP (Cell Signaling Technology, 1:100), cleaved caspase-3 (Cell Signaling Technology, 1:100), Bcl-2 (Cell Signaling Technology, 1:100), Bax (Cell Signaling Technology, 1:200) and ki67 (Abcam, 1:200) primary antibodies were used for immunohistochemistry according to a standard avidin-biotin complex (ABC) method. The sections were then incubated with appropriate biotinylated secondary antibodies provided in the ABC kit (PV-6000D, Beijing Zhongshan Jinqiao Biotechnology Co., Ltd) and visualized by exposure to diaminobenzidine substrate. The sections were finally counterstained with hematoxylin before dehydration, removal and sealing.

### Statistical analysis

All statistical calculations were performed using the GraphPad Prism 7 software package (GraphPad Software Inc., United States). Results were expressed as mean ± SD. Comparison between the control and CAP_PZ2_ treatment groups was achieved using the Student’s *t*-test, and *p* < 0.05 was defined as statistically significant (*p < 0.05; **p < 0.01; and ***p < 0.001).

## Results

### CAP_PZ2_ treatment duration-dependently inhibited proliferation and reduced viability of HT29 and A549 cells

**FIGURE 2:**
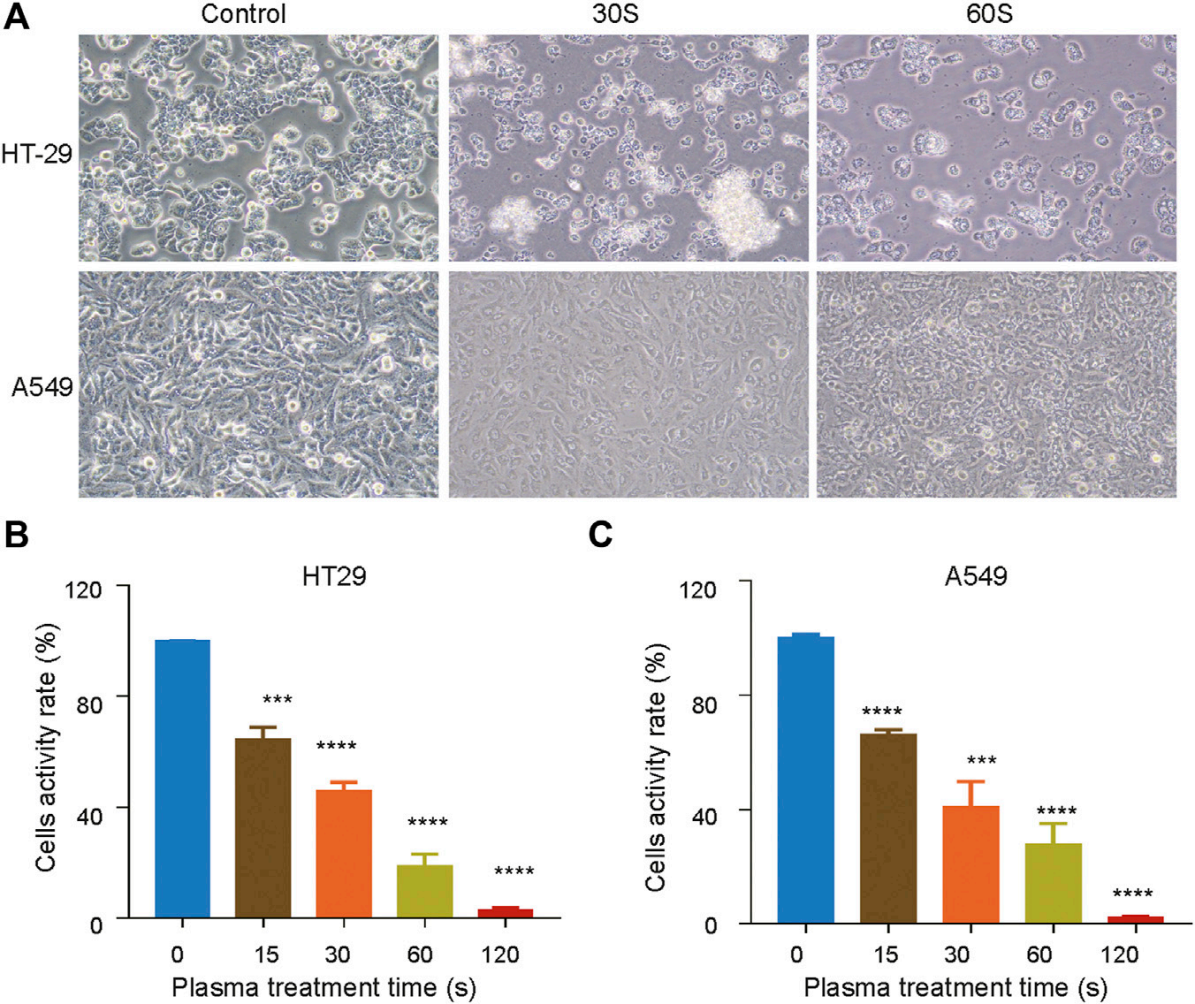
Inhibition of cell proliferation by CAP_PZ2_ treatment. **(A)** Optical images of the HT29 and A549 cellular damage caused by CAP_PZ2_ treatment. **(B, C)** Cell activity decreases while CAP_PZ2_ treatment duration increases. The above images and values represent results from three independent experiments and expressed as mean ± SD. *p < 0.05 was considered statistically significant.

The effect of CAP_PZ2_ (Tinsley and Harris, 2020) on cell viability and cell proliferation was measured using CCK8 assay. As the duration of plasma treatment increased (from 15 to 120 s), the viability of both HT29 (Figure 2B) and A549 (Figure 2C) cells decreased, exhibiting a duration-dependent manner. Using the current plasma parameters, most cancer cells had died by the end of 2 min plasma exposure. In addition, there appeared to be obvious morphological change in the tested tumor cells under phase contrast microscopy examination (Figure 2A). When the cells were exposed under CAP for 30 s, they looked more shrunk and less adherent to the cell culture dish, comparing to those before the plasma treatment. After the treatment duration was doubled, the HT29 and A549 cells became spherical and shriveled, and visually suspended in the cell culture medium, suggesting more prominent cell detachment.

### CAP_PZ2_ treatment decreased the migration and invasion of colorectal and lung tumor cell *in vitro*

**FIGURE 3:**
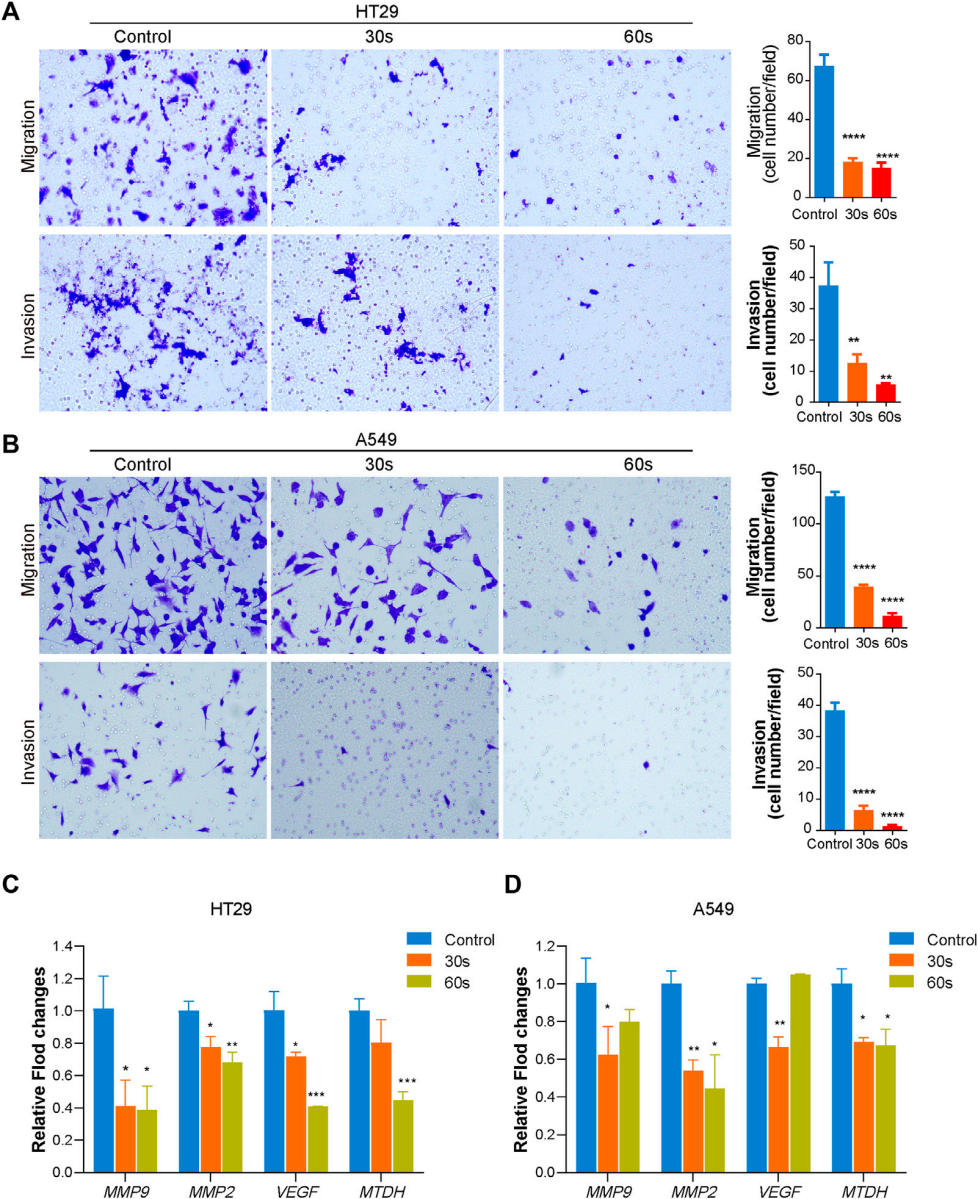
Effect of CAP_PZ2_ treatment on the migratory and invasive ability of the HT29 and A549 cells. The HT29 **(A)** and A549 **(B)** cells were treated by CAP_PZ2_ for 30 and 60 s, incubated for 24 h, before subjected to Transwell migration and Matrigel invasion assays. Representative images (left) and statistics (right) of migration and invasion assay were shown. The scale bar equals 200 μm. The experiments were performed in triplicates. **(C,D)** mRNA (*MMP9*, *MMP2*, *VEGF*, *MTDH*) expression changes during CAP_PZ2_ treatment with different time. *p < 0.05, **p < 0.01, and ***p < 0.001 versus control, *n* = 3.

The effect of CAP_PZ2_ on the *in vitro* tumor cell migration and invasion was investigated using the Transwell migration and Matrigel invasion assay, respectively. Under light microscope, the crystal violet stained HT29 cells (Figure 3A) and A549 cells (Figure 3B) both demonstrated reduced motility after plasma treatment. Plasma-induced suppression on the invasive and migratory capability of these cancer cells seemed to be more dramatic as treatment duration increased from 30 to 60 s.

As shown in Figures 3C,D, CAP_PZ2_ treatment significantly decreased metastatic gene expression in HT29 and A549 cells. Specifically, mRNA expression of *MMP9*, *MMP2*, *VEGF* and *MTDH* in HT29 cells decreased after 30 and 60 s CAP_PZ2_ treatment as a time dependent when compared to the untreated control (Figure 3C). A time dependent downregulation was observed for the expressions of genes *MMP2* and *MTDH* in A549 cells as well. In addition, the expressions of genes *MMP9* and *VEGF* were also downregulated after 30 s CAP treatment in A549 cells (Figure 3D).

### CAP_PZ2_ treatment induced apoptosis in HT29 and A549 cells

**FIGURE 4:**
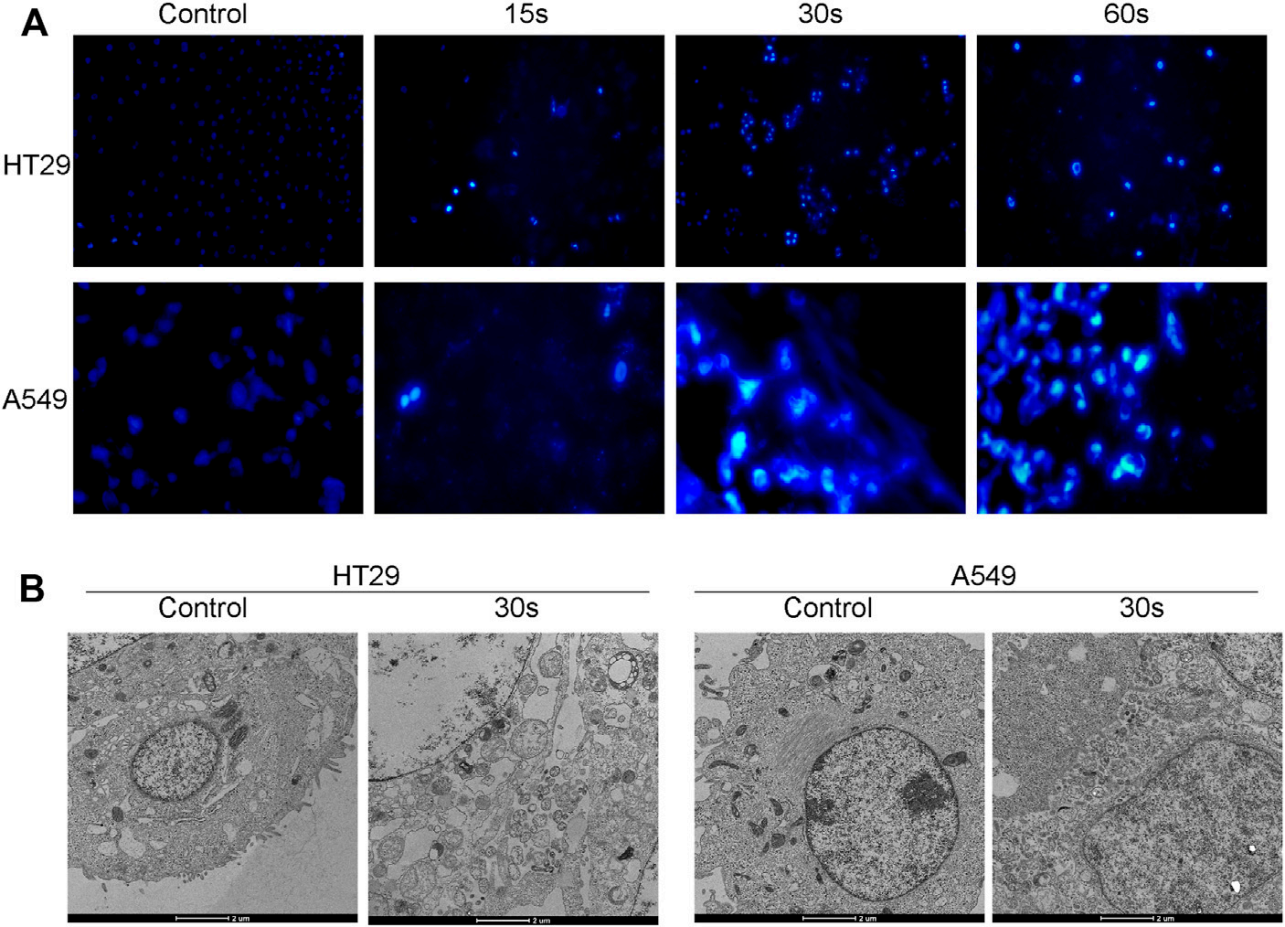
Qualitative analysis of the HT29 and A549 cellular apoptosis upon CAP_PZ2_ treatment. **(A)** Fluorescent microscopic images of nuclear changes (e.g., chromatin condensation) in the HT29 and A549 cells stained with Hoechst33342. **(B)** Transmission electron microscopic images of ultrathin sections (x10,000) demonstrating subcellular changes in the HT29 and A549 cells treated with CAP_PZ2_ for 30 s. All images are at the same magnification.

Apoptosis is a form of programmed cell death, which occurs in physiological and pathological conditions (Lv et al., 2008). In addition, apoptosis plays a pivotal role in tumorigenesis, tumor metastasis, and resistance to antineoplastic medications. Therefore, inducing apoptotic cell death is a promising strategy to prevent and treat cancer. In order to understand the mechanism of plasma-induced growth inhibition, the HT29 and A549 cells treated with CAP_PZ2_ were nuclei-stained and observed under fluorescent microscopy. As Figure 4A demonstrated, cells that were exposed to CAP_PZ2_ (15, 30 and 60 s) all had condensation of chromatin and appearance of apoptotic bodies, whereas untreated cells exhibited a typically non-adherent and round morphology. In order to gain further insight into the antiproliferative effect of CAP_PZ2_, transmission electron microscopy (TEM) was used to obtain ultrastructural information (Figure 4B). After the HT29 and A549 cells were treated with CAP_PZ2_ for 30 s, the TEM images discovered membrane damage, such as cell membrane blistering, nuclear membrane atrophy and irregular shape. In addition, there were chromatin condensation and nuclear fragmentation in the nuclei, as well as vacuolization and abnormal increase of mitochondria in the cytoplasm.

**FIGURE 5:**
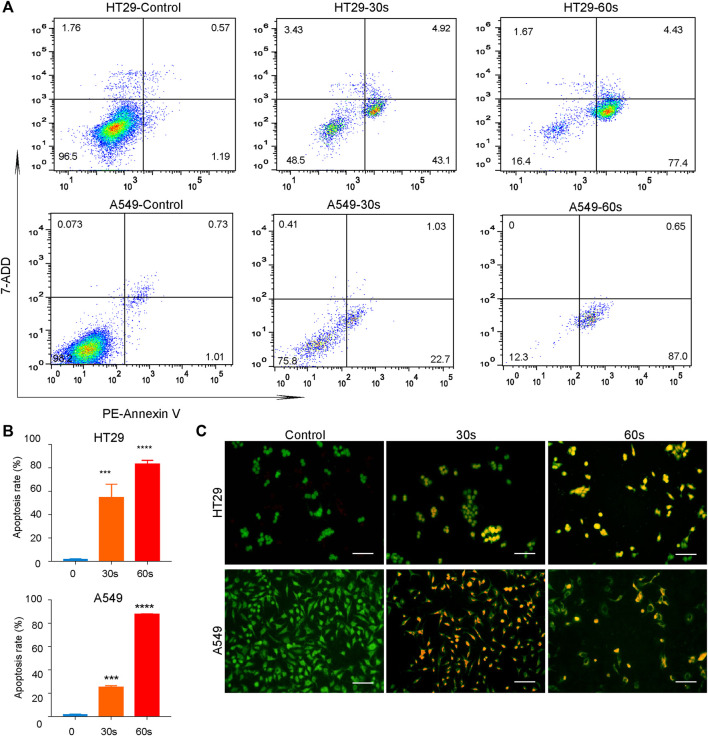
Quantitative analysis of the HT29 and A549 cellular apoptosis upon CAP_PZ2_ treatment. **(A)** Flow cytometric screening of the HT29 and A549 cells treated by CAP_PZ2_ for 30 and 60 s. PE Annexin-V/7-ADD staining was used to measure apoptosis rate. The proportion of cells in each quadrant are marked on the figures. **(B)** Histogram of the corresponding apoptosis rate expressed as mean ± SD of three independent experiments (***p < 0.001 and ****p < 0.0001). **(C)** Fluorescence microscopic images of the apoptotic HT29 and A549 cells detected using AO/EB staining. Green represents viable cells and orange represents apoptotic cells.

In addition to the above qualitative assessment, quantitative analysis of cellular apoptosis after CAP_PZ2_ treatment was conducted using flow cytometry. As shown in Figure 5A, PE annexin-V positive cells were considered as populations in the early and late apoptosis. CAP_PZ2_ induced a significantly increased population of apoptotic HT29 and A549 cells when compared with the negative control. This plasma-induced increase in the apoptosis rate seemed to be in a plasma dose-dependent manner (Figure 5B). This change in apoptosis was further evaluated through AO/EB staining. The HT29 and A549 cells were stained by AO/EB and analyzed using fluorescent microscopy (Figure 5C). Acridine orange is a vital dye that stains both living and dead cells, whereas ethidium bromide only stains cells that have lost their membrane integrity. Living cells turn green and can thus be distinguished from apoptotic cells (Ralph et al., 2016; Aranha et al., 2021). After treatment with CAP_PZ2_ for 30 and 60 s, a significant decrease in the viability of HT29 and A549 cells was detected, while the number of apoptotic cells increased after 30 and 60 s of plasma treatment. Collectively, these results indicate that tumor cell death after CAP_PZ2_ treatment occurred mainly through enhanced apoptosis.

### CAP_PZ2_ treatment raised intracellular reactive oxygen species accumulation

**FIGURE 6:**
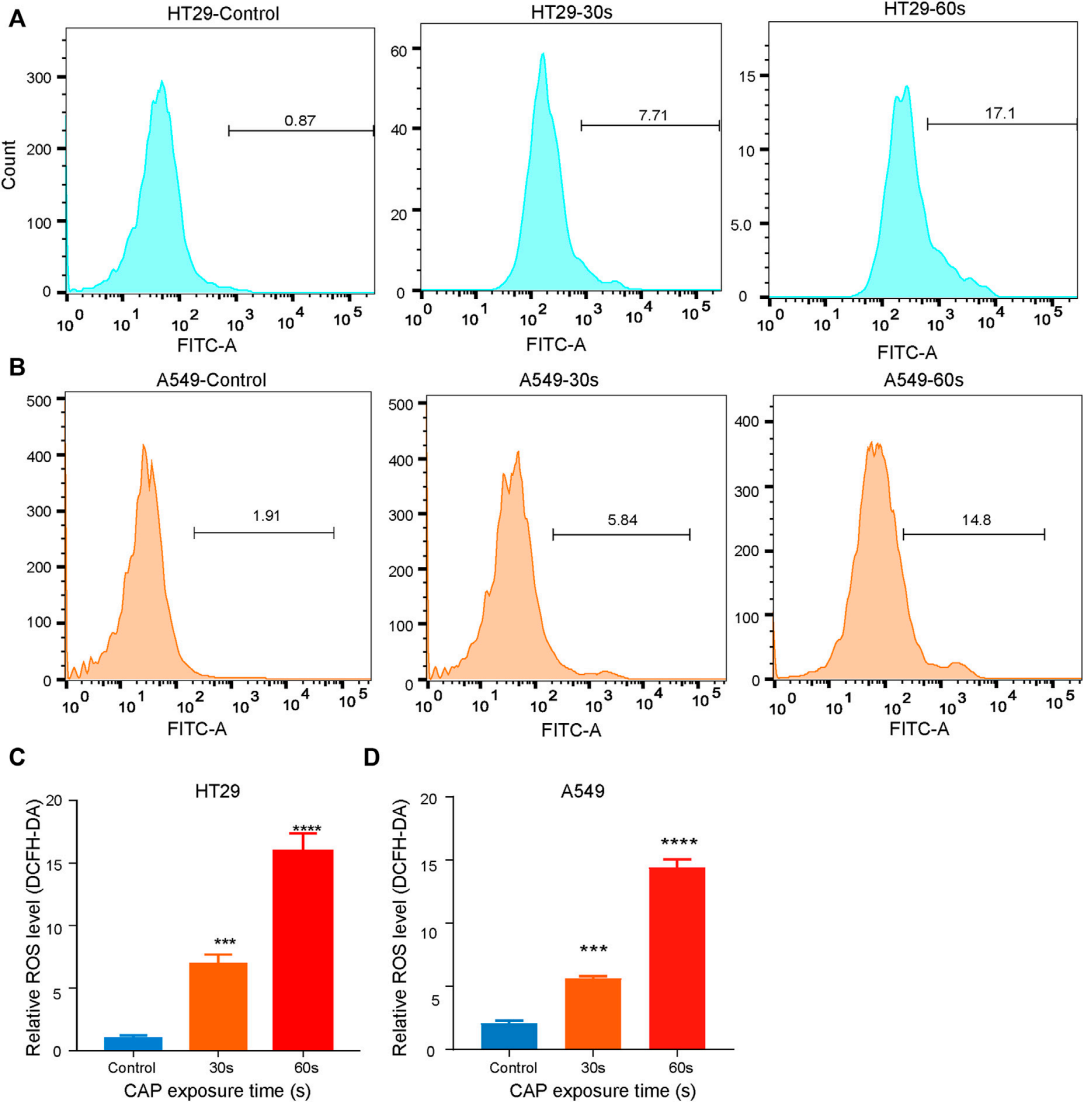
Dose-dependent and CAP_PZ2_-induced ROS accumulation in the HT29 and A549 cells. **(A, B)** Flow cytometric analysis of intracellular ROS after DCFH-DA incubation with cells treated using CAP_PZ2_ at 30 and 60 s. **(C, D)** Histogram showing the proportion of ROS negative cells after different plasma treatment duration. Student’s t-test was performed (***p < 0.001 and ****p < 0.0001).

The release of reactive oxygen species (ROS) by most apoptosis inducers is considered the key mediator of apoptosis signaling transduction (Negrin et al., 2010). Since increased ROS production in tumor cells may lead to the activation of mitochondrial pathways and cell death, we decided to investigate whether ROS are involved in CAP_PZ2_-induced apoptosis. As shown in Figure 6, the HT29 and A549 cells exposed to CAP_PZ2_ (30 and 60 s) exhibited a significantly enhanced accumulation of intracellular ROS (Figures 6A,B). When compared to untreated cells, the HT29 and A549 cells treated with 30 and 60s of CAP_PZ2_ demonstrated notable increase of fluorescence emitted by the ROS-induced oxidation of DCFH-DA (Figures 6C,D).

### Potential molecular pathway of plasma-induced apoptosis in HT29 and A549 cells

**FIGURE 7:**
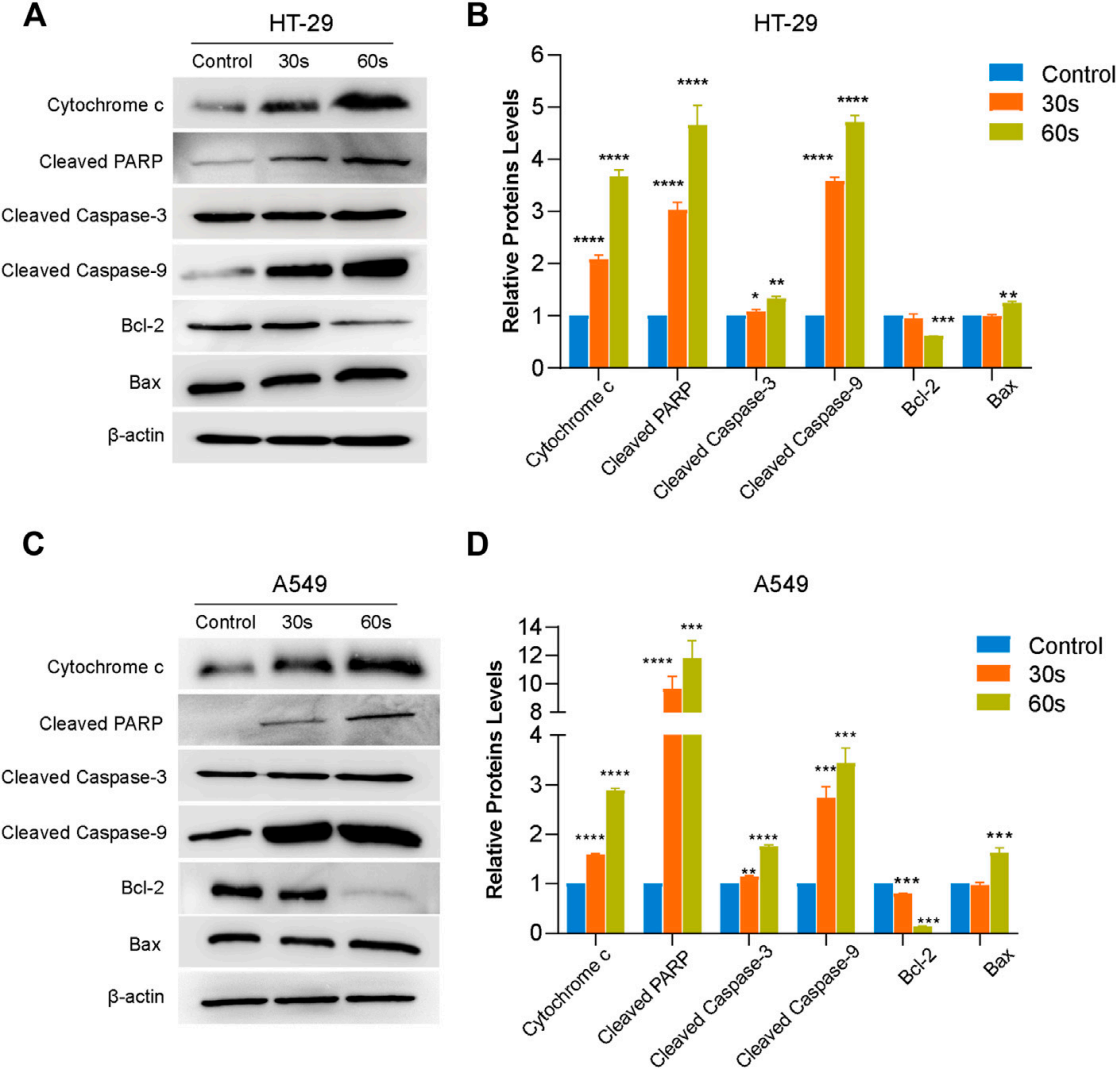
Western blot analysis revealing the potential signaling pathway of plasma-induced apoptosis in the HT29 and A549 cells treated using CAP_PZ2_. Proteins were extracted from the HT29 **(A,B)** and A549 **(C,D)** cells upon treatment by CAP_PZ2_ for 30 and 60 s, and subjected to Western blotting using the respective antibodies with β-actin antibody acting as a parallel blotting.*p < 0.05, **p < 0.01, ***p < 0.001 and ****p < 0.0001 versus control, *n* = 3.

In order to establish whether CAP_PZ2_ induces apoptosis through the mitochondrial pathway, we performed Western blot on relevant proteins that are differentially expressed by the HT29 and A549 cells treated with or without CAP_PZ2_. Firstly, as shown in Figures 7A–D, CAP_PZ2_ greatly increased the protein expression level of cytosol cytochrome-*c* when compared with the control group. Cytochrome-*c* is one of the most prominent actors in the apoptotic scene. Secondly, in order to further address the apoptotic effect of CAP_PZ2_ on the HT29 and A549 cells, we analyzed the levels of cleaved caspase-3, caspase-9 and cleaved PARP. PARP, a substrate of caspase-3, is an early hallmark of apoptosis. After plasma treatment for 30 and 60 s, the amount of cleaved PARP elevated with increasing plasma dose of CAP_PZ2_ treatment. The production of cleaved caspase-3 and caspase-9 also increased significantly in a plasma dose-dependent manner in the HT29 and A549 cells treated with CAP_PZ2_. Lastly, in order to investigate the molecular events involved in CAP_PZ2_-induced apoptosis, expression of the Bcl-2 and Bax rheostat that regulates antioxidant pathway and cell death was also assessed using Western blotting. The results showed that the expression of anti-apoptotic protein Bcl-2 was plasma dose-dependently decreased in the CAP_PZ2_ groups, whereas that of pro-apoptotic protein Bax was increased accordingly (Figure 7).

### CAP_PZ2_ treatment inhibited *in vivo* tumor growth in a xenograft mouse model

**FIGURE 8:**
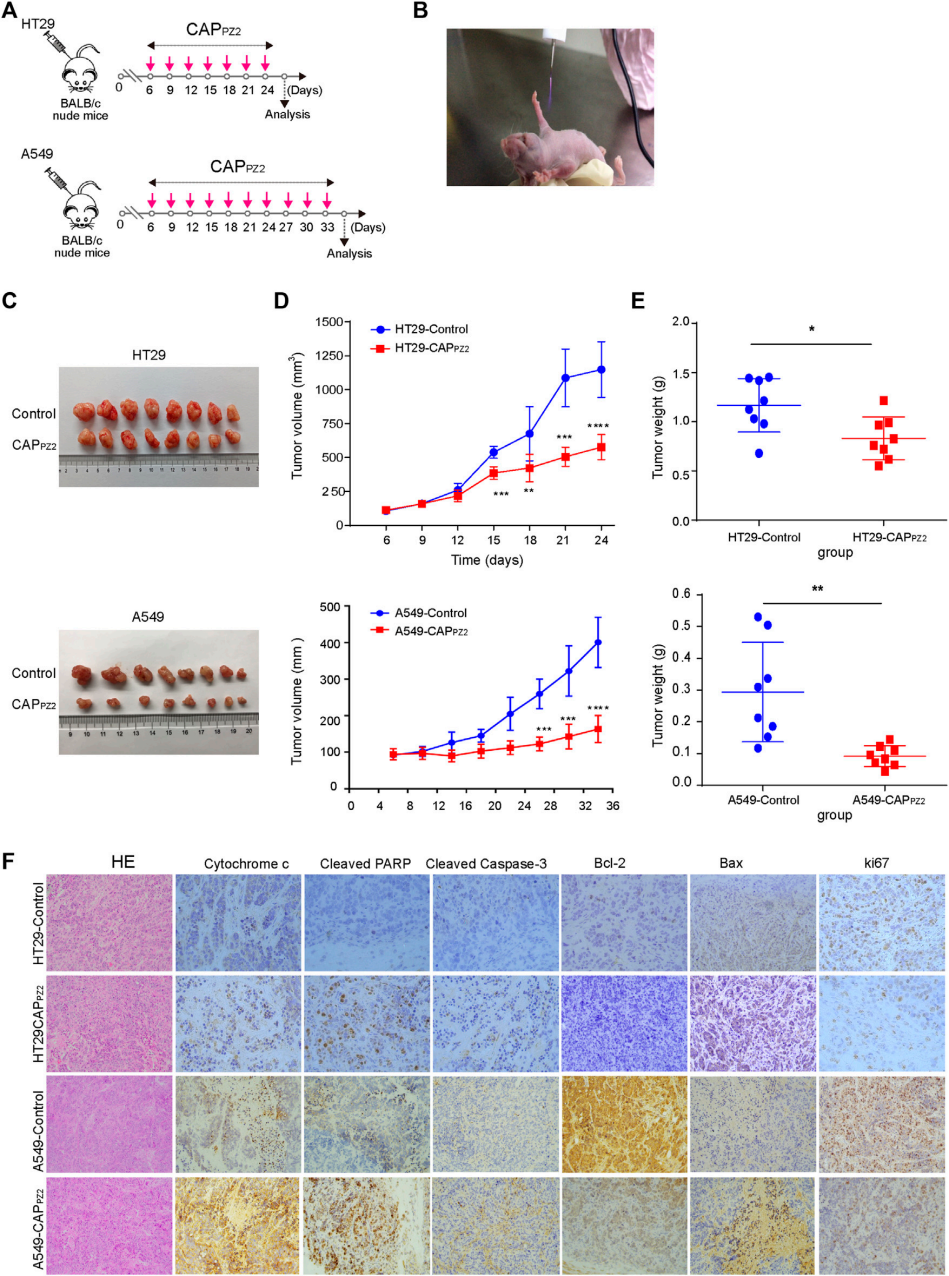
*In vivo* CAP_PZ2_ treatment of mouse xenograft tumors. **(A)** Flowchart of *in vivo* CAP_PZ2_ therapy regimen. **(B)** Photograph of an operating Piezobrush^®^ PZ2 CAP device irradiating tumors on a nude mouse bearing the HT29 or A549 cells. **(C)** Pathological specimens of resected *in situ* tumors with and without CAP_PZ2_ treatment. **(D)** Tumor growth curves of mice xenografts during the entire course of CAP_PZ2_ treatment. **(E)** Tumor weight after the last dose of plasma administration (*p < 0.05, **p < 0.01, ***p < 0.001, *n* = 8). **(F)** Representative histological images characterizing tumours and Immunohistochemical analysis using antibodies against various apoptosis-related proteins. Scale bar = 50 μm.

In order to verify the anti-neoplastic effect of CAP_PZ2_ *in vivo*, we performed a proof-of-principle experiment using a mouse xenograft model incorporating the HT29 or A549 cells (Figures 8A,B). The results collectively showed that the tumor growth was significantly suppressed in the CAP_PZ2_ treatment group. The qualitative photos of the excised tumors demonstrated that plasma-irradiated tumors appeared to be smaller than the untreated specimens (Figure 8C), especially at time points towards the end of experiment. On the other hand, the quantitative tumor measurement revealed that while the *in-situ* cancer grew steadily over time in the untreated group, tumor growth in the plasma-treated group appeared much slower in comparison (Figure 8D). The tumor volume in the A549 cells inoculated mice was virtually unchanged in the first few weeks. In addition, at a chose time point, the average tumor weight was significantly lower in the CAP_PZ2_-treated mice (Figure 8E).

In order to explore and verify the possible molecular mechanism involved in the *in vivo* oncological CAP_PZ2_ treatment, the expression of apoptosis-related proteins, such as cytochrome-*c*, cleavage-PARP, cleaved caspase-3, Bcl-2, Bax and ki67 proteins in the CAP_PZ2_-treated and control samples was analyzed using immunohistochemical staining. In our study, microscopic examination showed that the percentage of cytochrome-*c*, cleavage-PARP, cleaved caspase-3 and Bax positive cells was higher in the CAP_PZ2_-treated group compared to the control (Figure 8F). The apoptosis-promoting effect of CAP_PZ2_ was further confirmed by the results generated by IHC staining of Bcl-2. Majority of tumors from the control group showed moderate to strong staining intensity of Bcl-2, whereas the CAP_PZ2_-treated tumors presented weak or undetectable Bcl-2 expression (Figure 8F). This coincided with the reduction in tumor growth as indicated by the Ki67 staining. These results collectively revealed that the CAP_PZ2_-induced tumor inhibition may be through promotion of apoptosis, which is in line with the activation of cytochrome-*c*, PARP, cleaved caspase-3 and Bax, as well as downregulation of Bcl-2 and ki67 in the CAP_PZ2_-treated tumor samples.

## Discussion

Cancer is the leading cause of death in the developed countries and the second leading in the developing world. However, the non-surgical treatment options for solid malignant tumors have remained limited over the recent decades. Therapeutic modalities, such as radiation, chemotherapy, immunotherapy, administered alone or in combination, all have suboptimal therapeutic effects. Cold atmospheric plasma has emerged as a promising antineoplastic adjunct in recent years (Ratovitski et al., 2014). There have been copious preclinical and clinical reports pertaining CAP’s oncological application in various types of cancer, such as cutaneous, breast, liver, ovarian, pancreatic, oral, and thyroid cancers (Chang et al., 2014a; Chang et al., 2014b; Gay-Mimbrera et al., 2016; Dai et al., 2018). CAP essentially refers to a partially ionized gas containing reactive oxygen and nitrogen species, ions and electrons, and ultraviolet photons. This combination can bring physicochemical cues to biological targets without causing thermal damage to adjacent healthy recipients (Adhikari et al., 2019).

Although the mechanistic exploration of plasma oncology is still at a relatively early stage, several cellular events and molecular pathways have been discovered and proposed (Semmler et al., 2020). Previous studies have confirmed that appropriate dose of CAP usually does not cause cell necrosis, but rather sublethal or lethal cellular reactions (e.g., detachment and apoptosis) in various cell types (Wang et al., 2011; Adhikari and Arora, 2015; Zhu et al., 2016). Normal tissue homeostasis and cell function are maintained by the balance between proliferation and apoptosis (Wang et al., 2012). Cancer is a typical disease in which malignant cell clones escape this equilibrium and improperly proliferate without compensatory apoptosis (Kim et al., 2007). In other words, premalignant cells grow faster than normal cells do because of the imbalance of cell growth and cell death mechanisms. Therefore, the success of a cancer treatment largely depends on the degree to which they preferentially induce tumor cell death while allowing normal tissue to survive.

According to the latest global cancer burden data released by the International Agency for Research on Cancer under WHO in 2020, the incidence rate of lung cancer and colon cancer ranks top two and three, with the former still being the most lethal cancer worldwide. Therefore, we used colon cancer cells HT29 and lung cancer cells A549 as the *in vitro* experimental model in this study. Our preliminary results showed that CAP_PZ2_ treatment inhibited proliferation of the above cells in a plasma dose-dependent manner and caused morphological changes, such as cell shrinkage, chromatin condensation and nuclear fragmentation. These results are consistent with independent studies from other research groups using different cancer cell models (Xia et al., 2019; Bekeschus et al., 2020).

Cancer cell invasion and migration are the major differentiating features of malignant tumors from benign ones. They are also one of the important causes of treatment failure. Thus, preventing migration and invasion of cancer cells and associated tumor metastasis is a hotspot in cancer research. Cancer cell migration is usually regulated by adhesion receptors, matrix-degrading enzymes, and cell-cell adhesion molecules. However, even cancer therapeutics specifically targeting these regulators sometimes prove ineffective in clinical trials, mainly because cancer cells can modify their migrative mechanism in response to various conditions (Friedl and Wolf, 2003). Fortunately, plenty of studies have shown that CAP treatment can effectively inhibit tumor invasion and metastasis, which provides a novel strategy for cancer therapy (Chang et al., 2014b; Li et al., 2016). Our results were consistent with other reports (Kang et al., 2017), since the migration and invasion of HT29 and A549 cells were significantly inhibited by CAP_PZ2_ treatment. MMP9 and MMP2 have similar biological function and they play a key role in the decomposition of extracellular matrix. During the development of cancer, MMP9 and MMP2 degrade the basement membrane and promote the metastasis of tumor cell to distant tissues and/or organs. VEGF is a protein that stimulates the growth and formation of the circulatory system and blood vessels (angiogenesis and angiogenesis). Regarding its role in cancer, VEGF promotes the presence of nutrients and oxygen in cancer cells. More blood supply promotes cancer cell proliferation and inhibits apoptosis. Therefore, downregulation of VEGF expression could be used as an indicator of inhibiting tumor progression. A more direct indicator of cancer progression, MTDH is an oncogene which promotes cancer cell proliferation. Moreover, MTDH is attributed to the development of chemoresistance as well as increasing metastatic potential. In our study, we found CAP_PZ2_ treatment downregulated all four markers in HT29 and A549 cells. In addition, since ROS are considered to have important roles during cancer cell migration and invasion (Tochhawng et al., 2013), and CAP happens to generate extracellular and intracellular ROS, further study to explore the role of plasma-mediated redox balance for CAP’s anti-invasion and anti-migration effects are warranted.

Our further study exploring the cellular events underlying CAP’s inhibitory effect on HT29 and A549 cell proliferation discovered that the main contributing factor is increased rate of early apoptosis and late apoptosis in these cells after plasma treatment. These results were consolidated by morphological signs of apoptosis under microscopic observation. Other studies have also reported similar findings on CAP’s antiproliferative effect and CAP-induced apoptosis (Zhong et al., 2016; Gan et al., 2019). Apoptosis, the process of programmed cell death, plays a pivotal role in the pathogenesis of many diseases. Apoptosis is characterized by distinct morphological features and regulated by a series of biochemical events leading to cell death (Elmore, 2007). Cancer is one of the situations where too little apoptosis happens, leading to reduced death in malignant cells. Mechanistic insights into the impact of CAP-induced apoptosis have been a hot area of research in plasma oncology.

Recent studies revealed that CAP can trigger RONS-based tumor cell apoptosis (Bauer et al., 2019). RONS has been shown to induce various biological processes, including apoptosis. This means that the redox state of cells is a key factor to determine their sensitivity to apoptotic stimulation (Chen et al., 2015). Low concentration of ROS plays a role of intracellular messenger in many molecular events, such as cell proliferation, whereas high density of ROS contributes to cell apoptosis. Our results demonstrated a dramatic burst of ROS in a plasma dose-dependent fashion after CAP_PZ2_ treatment, suggesting that ROS generation is involved in the CAP_PZ2_-induced apoptosis in HT29 and A549 cells. Dezest et al. (2017) found that the proteasome, a crucial intracellular proteolytic system which modulate tumor cell growth and survival, is a target of CAP. Interestingly, in addition to RONS, plasma-generated charged particles and electromagnetic emission also play an important role in cancer cell death (Yan et al., 2020).

Since we have depicted the cellular events and intracellular chemical cues involved in the *in vitro* cancer treatment by CAP, we performed further study to explore the potential molecular pathway during CAP_PZ2_-induced apoptosis. Apoptosis can be initiated by a death receptor-mediated pathway or a mitochondria-mediated pathway (Wang et al., 2014). In the mitochondria-mediated pathway, cytochrome *c* is released from mitochondria to cytoplasm due to the change of mitochondrial membrane permeability. The ROS produced by CAP treatment triggers molecular signaling pathways and promotes mitochondrial disturbance, resulting in apoptosis (Ahn et al., 2014). To further assess the anti-tumor mechanism of CAP_PZ2_, we discovered that CAP_PZ2_ treatment greatly increased cytochrome *c* in cytosol of HT29 and A549 cells, suggesting that the intrinsic apoptosis route *via* mitochondria is involved. Once cytochrome *c* is released, it forms apoptotic bodies with Apaf-1 and procaspase-9 in the presence of dATP, leading to activation of caspase-9. Caspases play an important role in the executive stage of apoptosis (Wang et al., 2012). In the death receptor-mediated pathway, caspase-9 is activated downstream of the death-inducing signaling complex. The active caspase-9 activates downstream caspase-3, which leads to activation of the mitochondrial pathway (Cooper et al., 2007). The effector caspases may cleave a number of structural and regulatory cellular proteins including PARP and lamin protein, and are responsible for the typical morphological and biochemical features of an apoptotic cell (Bossy-Wetzel and Green, 1999).

The mitochondrial pathway is one of the most important cellular apoptosis signal transduction pathways, and Bcl-2 family is a key regulatory factor of this pathway. It can alter the balance between pro-apoptotic and anti-apoptotic pathway in tumor cells, determine the response of cells to external stimuli, and thus determine the fate of cells (Turrini et al., 2017). As an anti-apoptotic factor, Bcl-2 maintains the integrity of mitochondrial membrane, while Bax promotes apoptosis by destroying the mitochondrial membrane (Antonsson, 2004). Our results showed that the effect of CAP_PZ2_ treatment did cause these events through apoptosis, as CAP_PZ2_ markedly elevated the expression of cleaved form of PARP, caspase-3, and caspase-9. Moreover, we have also found an increase in the expression of Bax protein and a decrease in the expression of Bcl-2 in HT29 and A549 cells treated using CAP_PZ2_. An increase in the ratio of Bax/Bcl-2 stimulates the release of cytosolic cytochrome *c*, leading to the activation of caspase-3 and PARP. These results further confirmed that CAP_PZ2_ oncotherapy can promote apoptosis of the HT29 and A549 cells through caspase-3 modulation *via* the mitochondrial pathway.

The number of other mechanistic studies exploring the molecular pathway of plasma-induced cancer cell apoptosis are still limited. Xia et al. (2019) reported that CAP induces apoptosis of melanoma cells *via* Sestrin2-mediated nitric oxide synthase signalling. Haralambiev et al. (2020) discovered that CAP treatment of osteosarcoma cells resulted in suppressed cell growth, activation of caspase-3/caspase-7 and degradation of genomic DNA. This apoptotic effect seemed independent of the plasma devices used. Yang et al. (2020) demonstrated that CAP-activated solution induces apoptotic signaling in basal cell carcinoma cells, and that this effect was associated with the activation of the MAPK signaling pathway. Finally, Estarabadi et al. (2021) found that both direct and indirect CAP treatment could not only induce cytotoxicity by shifting Bax/Bcl-2 towards apoptosis, but also cause genotoxicity in oesophageal cancer cells. Although there has been research reporting that synergistic treatment using CAP and silymarin nanoemulsion could inhibit melanoma tumorigenesis *via* targeting HGF/c-MET downstream pathway (Adhikari et al., 2019), our discussion here is limited to standalone CAP treatment. Over the last several years, a mainstay and goal of clinical plasma oncology has been the exploration of the mechanism of plasma-triggered cancer cell apoptosis. However, this is facing considerable challenge partially because the interaction of apoptosis pathways with other signalling mechanisms can also affect cell death. Once we have gained better understanding in this area of research, CAP has the potential to serve as a customizable adjuvant therapy for various types of cancer.

As we have successfully applied CAP_PZ2_ treatment *in vitro* for lung cancer and colon cancer cells, we furthered our work by exploring its therapeutic potential using cell-derived xenograft (CDX) models in animals. Our results showed that CAP_PZ2_ could significantly inhibit the growth of tumors *in vivo*. Immunohistochemically, in the tumors developed in these mice, the percentages of cytochrome-*c*, cleavage-PARP, and cleaved caspase-3 positive cells were higher in the CAP_PZ2_-treated group. Further results on Bax/Bcl-2 coincided with the reduction in tumor growth as indicated by the Ki67 staining. In other word, it is most likely that the anticancer effect on HT29 or A549 cells xenografted mice was also due to CAP_PZ2_-induced apoptosis through the mitochondrial pathway. So far, the CAP induced-apoptosis studies using various cancer cell lines *in vitro* vastly outnumber those using *in vivo* models. Our near future plan includes establishing patient-derived xenograft (PDX) models for oncological purpose in plasma medicine. Our ultimate objective is to contribute to developing a versatile and effective CAP-based oncotherapy after meticulous clinical trials.

## Conclusion

In this preclinical study, we are the first to demonstrate universal anti-tumor effect on lung cancer and colon cancer cells models using cold atmospheric plasma. A handheld piezoelectric direct discharge CAP device, Piezobrush® PZ2, was used throughout this work. The anti-proliferative effect of CAP_PZ2_ was established using human colorectal adenocarcinoma HT29 cells and human lung adenocarcinoma A549 cells *in vitro*. At the cellular level, CAP_PZ2_ treatment induced cancer cell apoptosis, inhibited tumor cell migration and invasion, and changed the morphological integrity and stability of mitochondria. At the protein and molecular level, CAP shifted the expression of apoptosis-related markers, such as Bcl-2, Bax, Cyt *c*, and caspase-3, towards the anti-apoptotic exit. On the other hand, the antineoplastic effect of CAP was confirmed *in vivo* using cell-derived xenograft animal models. The immunohistochemical analysis again revealed activation of Cyt *c*, PARP, cleaved caspase-3 and Bax, and suppression of Bcl-2 and ki67 in the plasma-treated specimens, which is consistent with *in vitro* results. Collectively, we revealed that CAP_PZ2_ could induce apoptosis of lung cancer and colon cancer cells *in vitro* and *in vivo* through the mitochondrial pathway.

## Data Availability

The original contributions presented in the study are included in the article/Supplementary Material, further inquiries can be directed to the corresponding author.
