# Supplementary material for: Cold atmospheric plasma induces apoptosis in human colon and lung cancer cells through modulating mitochondrial pathway
Source: Front Cell Dev Biol. 2022 Jul 26;10:915785. doi: 10.3389/fcell.2022.915785 (PMC9360593; doi:10.3389/fcell.2022.915785)
Supplement: Supplementary file 1 [file DataSheet1.PDF]

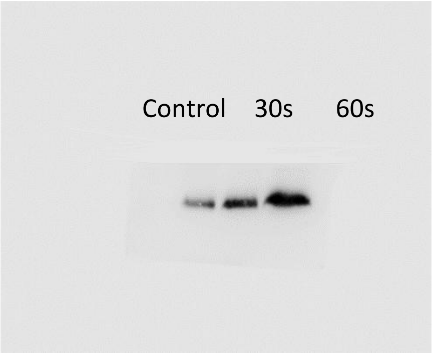

Fgiure 7A. HT29-Cytochrome *C*

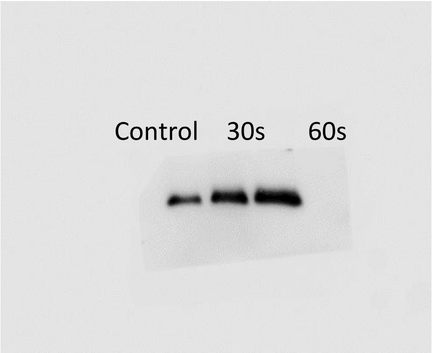

Fgiure 7B. A549-Cytochrome *C*

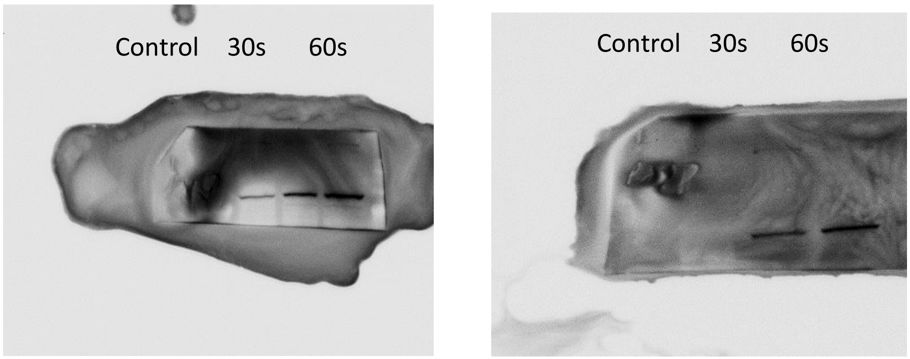

Fgiure 7A. H29-Cleaved PARP

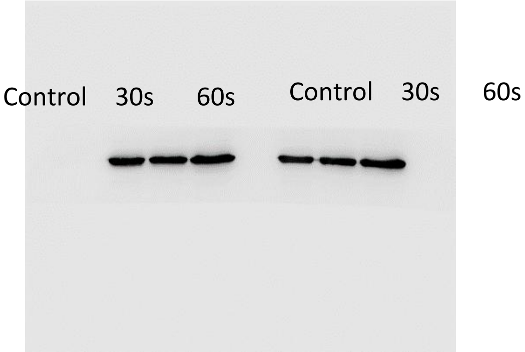

Fgiure 7A. H29-Cleaved Caspase-3 and B. A549-Cleaved Caspase-3

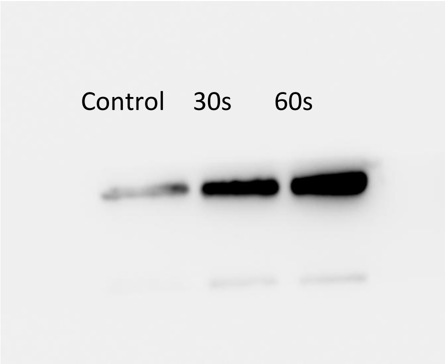

Figure 7A HT29-cleaved Caspase-9

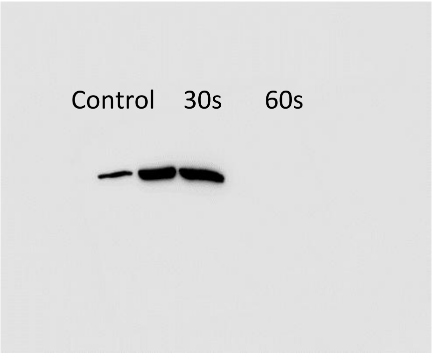

Figure 7B A549-cleaved Caspase-9

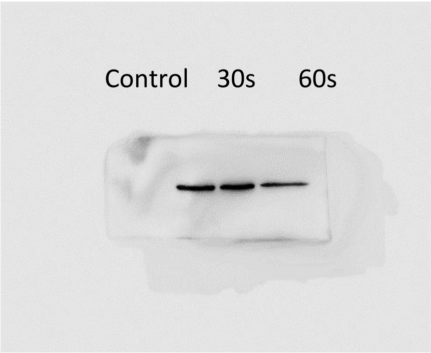

Figure 7A HT29-bcl2

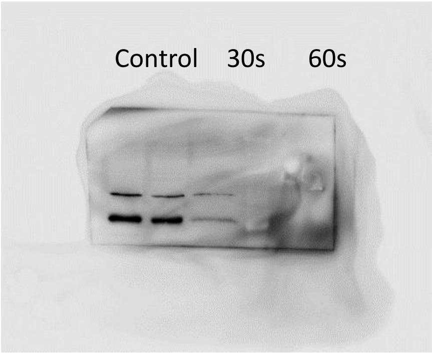

Figure 7B A549-bcl2

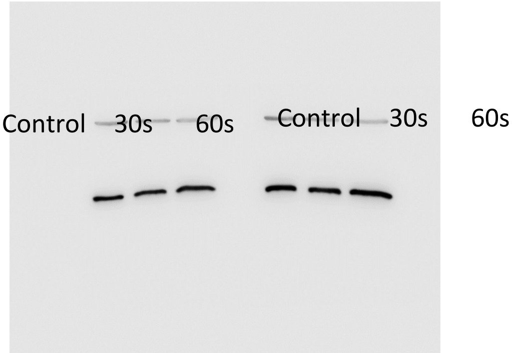

Fgiure 7Aand B. H29-Bax and A549-Bax

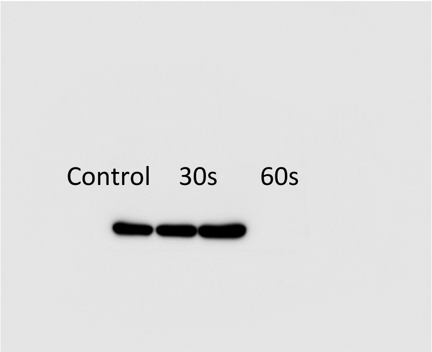

Figure 7A HT29-β-actin

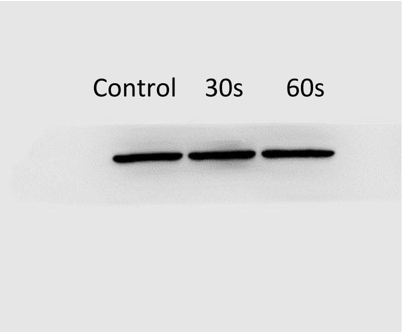

Figure 7B A549-β-actin
